# Characterization of immunoglobulin G antibodies to *Plasmodium falciparum *sporozoite surface antigen MB_2_ in malaria exposed individuals

**DOI:** 10.1186/1475-2875-8-235

**Published:** 2009-10-23

**Authors:** Thanh V Nguyen, John B Sacci, Patricia de la Vega, Chandy C John, Anthony A James, Angray S Kang

**Affiliations:** 1 Department of Molecular Biology and Biochemistry, University of California, Irvine, CA 92697, USA; 2 NeoGenomics California, 6 Morgan, Suite 150, Irvine, CA 92618, USA; 3 Department of Microbiology and Immunology, University of Maryland, School of Medicine, Room 324 660 W Redwood Street, Baltimore, MD 21201, USA; 4 Department of Cell Mediated Immunity, Division of Malaria Vaccine Development, US Military Malaria Vaccine Program, Walter Reed Army Institute of Research, USA; 5 Global Pediatrics Program and Division of Pediatric Infectious Diseases, University of MN Medical School, 420 Delaware Street, SE, MMC #296, 850-Mayo, Minneapolis, MN 55455, USA; 6 Department of Microbiology and Molecular Genetics, University of California, Irvine, CA 92697-3900, USA; 7 The School of Life Sciences, Department of Molecular and Applied Biosciences, University of Westminster, 115 New Cavendish Street, London, W1W 6UW, UK

## Abstract

**Background:**

MB2 protein is a sporozoite surface antigen on the human malaria parasite *Plasmodium falciparum*. MB2 was identified by screening a *P. falciparum *sporozoite cDNA expression library using immune sera from a protected donor immunized via the bites of *P. falciparum*-infected irradiated mosquitoes. It is not known whether natural exposure to *P. falciparum *also induces the anti-MB2 response and if this response differs from that in protected individuals immunized via the bites of *P. falciparum *infected irradiated mosquitoes. The anti-MB2 antibody response may be part of a robust protective response against the sporozoite.

**Methods:**

Fragments of polypeptide regions of MB2 were constructed as recombinant fusions sandwiched between glutathione S-transferase and a hexa histidine tag for bacterial expression. The hexa histidine tag affinity purified proteins were used to immunize rabbits and the polyclonal sera evaluated in an *in vitro *inhibition of sporozoite invasion assay. The proteins were also used in immunoblots with sera from a limited number of donors immunized via the bites of *P. falciparum *infected irradiated mosquitoes and plasma and serum obtained from naturally exposed individuals in Kenya.

**Results:**

Rabbit polyclonal antibodies targeting the non-repeat region of the basic domain of MB2 inhibited sporozoites entry into HepG2-A16 cells *in vitro*. Analysis of serum from five human volunteers that were immunized via the bites of *P. falciparum *infected irradiated mosquitoes that developed immunity and were completely protected against subsequent challenge with non-irradiated parasite also had detectable levels of antibody against MB2 basic domain. In contrast, in three volunteers not protected, anti-MB2 antibodies were below the level of detection. Sera from protected volunteers preferentially recognized a non-repeat region of the basic domain of MB2, whereas plasma from naturally-infected individuals also had antibodies that recognize regions of MB2 that contain a repeat motif in immunoblots. Sequence analysis of eleven field isolates and four laboratory strains showed that these antigenic regions of the basic domain of the *MB2 *gene are highly conserved in parasites obtained from different parts of the world. Moreover, anti-MB2 antibodies also were detected in the plasma of 83% of the individuals living in a malaria endemic area of Kenya (n = 41).

**Conclusion:**

A preliminary analysis of the human humoral response against MB2 indicates that it may be an additional highly conserved target for immune intervention at the pre-erythrocytic stage of *P. falciparum *life cycle.

## Background

Parasites of the *Plasmodium *species that are transmitted to people through the bites of infected mosquitoes cause malaria, a life-threatening disease. Malaria poses a serious public health problem in many parts of the world and approximately half of the world's population is at risk, in particular those living in lower-income countries [1]. The four types of human malaria are caused by *Plasmodium falciparum, Plasmodium vivax, Plasmodium malariae and Plasmodium ovale*. Of these, *P. falciparum *and *P. vivax *are the most common and *P. falciparum *is the most deadly [1]. Emergence of drug and insecticide resistance has exacerbated the situation, undermining the effectiveness of existing malaria control methods that depend on chemotherapy and vector control, respectively. Clearly, additional effective means to fight the disease, such as a safe and effective vaccine(s) are needed urgently. Currently, several approaches to developing malaria vaccine are in various stages of pre-clinical and clinical development involving single and multi-stage targets these are discussed in depth elsewhere [2-6].

Successful vaccination of humans on a limited scale against *P. falciparum *malaria was achieved first using irradiated sporozoites as an immunogen [7]. This approach follows the classical route of vaccine development via attenuation; in this case radiation induced attenuation resulting in non-replicating metabolically-active *P falciparum *sporozoites and results in targeting the pre-erythrocytic stage. This type of vaccine has to be 100% effective to induce sterile protective immunity and prevent the development of blood-stage infection in naïve individuals. Other vaccine candidates targeting the pre-erythrocytic stage that are less than 100% effective, may not prevent, but delay the onset of disease in naïve individuals and reduce subsequent episodes of clinical malaria [8], and as such may still play an important role in the fight against malaria. Although non-replicating metabolically-active sporozoites as immunogen(s) appears to be effective and the limited data are encouraging, the development of this approach leading to a licensed product for the prevention of malaria infection presents challenges and opportunities [9]. As efforts continue to develop this potential pre-erythrocytic stage attenuated vaccine, the volunteers that have already participated in the early phases of validation warrant further evaluation to examine the nature of this induced sterile protective response with a view to identifying key responsive elements to provide insights into the molecular basis of this immunity.

The pre-erythrocytic immune response is primarily directed against the circumsporozoite (CS) protein, a surface protein of *Plasmodium *sporozoites [10-12]. The CS protein is a leading vaccine candidate because irradiated sporozoite-induced protection in volunteers correlates with high circulating levels of anti-CS antibodies [13], and these antibodies are directed against the immunodominant B cell epitopes in the central tetramer repeat [Asparagine Proline Asparagine Alanine]_n _(NPNA)_n_. Moreover a human monoclonal antibody directed against CS protein (NPNA)_n _tetramer repeat isolated from a protected individual immunized via bites of infected, irradiated mosquitoes and subsequently shown to be protected against non-irradiated parasite challenge exhibited dose-dependent inhibition of *P. falciparum *sporozoites invasion of HepG2-A16 cells *in vitro *[14,15]. Recent attempts to induce protection in humans using *P. falciparum *CS-based vaccines containing the NPNA repeat motif, with improvement in their immunogenicity, and formulation, such as RTS, S/AS02 have yielded promising results [16]. To induce the protective immunity that is as consistent and long-lasting as that observed in the irradiated-sporozoite exposed individuals may require a vaccine that not only targets additional sporozoites surface antigens, but also provides a mechanism for extended priming of the immune system. The later present significant challenges in vaccine delivery and short of radiation or genetically-attenuated non-replicating metabolically-active sporozoite, may be difficult to mimic. However, in addition to the immunodominant CS protein it may be important to identify antigens that may act independently, additively or synergistically and contribute towards more robust protection. To-date several candidate molecules, including thrombospondin-related anonymous protein (TRAP)[17], also know as sporozoite surface protein 2 (SSP2)[18], sporozoite-threonine-asparagine-rich protein (STARP)[19] and the more recently identified MB2 [20], have been shown to be present on the sporozoite surface. The *P. falciparum *gene *MB2*, is a single copy gene on chromosome five, and is expressed as a single mRNA transcript in erythrocytic stage of parasites, the predicted translational product is 1610 amino acids in length with a predicted mass of 187 kDa. The translated primary sequence may be further subdivided into three domains, an amino terminal basic domain, a central acidic domain and a carboxyl terminal domain with high sequence similarity to GTP-binding domains. The *MB2 *gene products are present in the sporozoite, asexual blood stages and gametocytes and have a distinct pattern of stage dependent sub-cellular localisation and proteolytic processing at the various stages of the parasite life cycle[20]. To-date gene homologues to the *P. falciparum MB2, (PfMB2) *have been identified in *Plasmodium knowlesi(PkMB2), Plasmodium gallinaceum (PgMB2), Plasmodium berghei (PbMB2), Plasmodium yoelii (PyMB2), Plasmodium chabaudi (PcMB2) *[21] and *P. vivax(PvMB2) *(GenBank Accession XM_001613742). Of four conserved regions of the putative *MB2 *translation products, two have similarity to known proteins, S1 domain involved in initiation of translation and mRNA turnover and a GTP-binding domain similar to the family G-domains involved in protein synthesis [21]. In the non-conserved region of *PfMB2, PyMB2 *and *PgMB2 *tandemly repeated amino acid motifs are present. Repeat motifs are present in other malarial surface proteins including CS, TRAP and merozoite surface proteins (MSP) [22]. These repetive regions are T-cell independent repeat regions of parasite antigen and it has been postulated that the immune response against these regions diverts the response away from more important epitopes [23].

The MB2 antigen possesses intriguing immunogenic and molecular properties that indicate that it may be an important immune target in the non-replicating metabolically active *P. falciparum *sporozoite attenuated vaccine and may complement and enhance the efficacy of pre-erythrocytic stage vaccines currently in development.

## Methods

### Recombinant protein expression and purification

Fragments of the *MB2 *open reading frame were expressed in bacteria as Glutathione-S-Transferase (GST)-MB2-6xHistidine (His) fusion proteins in the dual-affinity pAK1-6H expression vector [24]. The oligonucleotide primers used to construct the GST-MB2-6xHis recombinant proteins are listed in Table 1. The cloning of *MB2 *fragments into the expression vector was described in earlier studies [20]. Purification of recombinant proteins was carried out using the ProBond resin (Invitrogen) with the addition of imidazole at 85 mM final concentration in the washing buffer. Eluted fractions were analysed by SDS-PAGE and immunoblotting using anti-GST antibodies.

**Table 1:** Names, positions, and sequences of oligonucleotide primers used to clone and express GST-fusion proteins representing various regions of the MB2 open reading frame.

**GST-fusion**	**Amino acid**^**a**^
**MB2-A**	
**5' primer-Nco I **^b^(98)GATG**CCATGGG**TGTTAATAGATGTTTTAC	
**3' primer-Sma I **(305)GAT**CCCGGG**GAGCATATTCTATTATATTCA	32 - 101
**MB2-B**	
**5' primer-Nco I **(286)GATG**CCATGG**AATATAATAGAATATGCA	
**3' primer-Sma I **(620)GAT**CCCGGG**TTTTTATTATTAGAAGAATCA	95 - 206
**MB2-C^**c**^**	
**5' primer-Nco I **(602)GATG**CCATGG**ATTCTTCTAATAATAAAAT	
**3' primer-Sma I **(953)ATGCAT**CCCCGGG**TCATTTTTTATTTGAAGAATTCTC	200- 316
**MB2-F^**d**^**	
**5' primer-Nco I **(98)GATG**CCATGG**GTGTTAATAGATGTTTTATC	
**3' primer-Sma I **(953)ATGCATC**CCCGGG**TCTTTTTTATTTGAAGAATTCTC	32 - 316
**MB2-D**	
**5' primer-Nco I **(1066)GATG**CCATGG**CATCTACATTAGATGAAACA	
**3' primer-Sma I **(1640)GAT**CCCGGG**GATGTACTATAATCATTATTTGG	355 - 546
**MB2-E**	
**5' primer-Nco I **(1616)GATG**CCATGG**ATCCAAATAATGATTATAGTACA	
**3' primer-Sma I **(2321)GAT**CCCGGG**CTTGAATTATATTCTTTATTTTCGTG	538 - 773
**MB2-FA^**c**^**	
**5' primer-Nco I **(2294)GTATG**CCATGG**TCCACGAAAATAAAGAATATAATTCAAG	
**3' primer-Sma I **(2837)GAT**CCCGGG**TCATCGAGCGATTCATTTTGGTC	764 - 945
**MB2-IF2**	
**5' primer-Nco I **(4009)GATG**CCATGG**ATGGTAATAGAACAAATAATGAC	
**3' primer-Sma I **(4823)GAT**CCCGGG**TACGCTTCGATTATATCGTTTGGCTC	1337 - 1606

^a ^Numbers indicate amino acid positions relative to the initiation methionine (1), ^b ^Numbers in parenthesis refer to nucleotide positions relative to the ATG initiation codon; bold underlined letters indicate the restriction endonuclease sites, *Nco *I and *Sma *I,^c ^These peptides includes amino acid repeats, ^d ^This peptide is a combination of MB2-A, -B, and -C.

### Human plasma and serum

Samples of plasma were obtained from individuals living in an area of Kenya with highly seasonal malaria transmission. Samples were obtained during high transmission season from individuals two years of age or older. Plasma donors were assessed for symptoms of malaria and the presence of *P. falciparum *parasitaemia on microscopic examination of peripheral blood smears. Of the forty-one plasma donors, nine were smear negative, seven were smear positive but asymptomatic, and twenty-five were smear positive and symptomatic. Symptomatic individuals had one or more of the following symptoms: fever, chills, headache, severe malaise or vomiting. Samples with available serum were tested for antibodies to CSP and TRAP by ELISA, as described previously [25]. Levels were given in arbitrary units (AU). The AU was determined by dividing the optical density (OD) of the study sample by the mean plus three standard deviations of the ODs of nine North American control sera. The cut-off for a positive response was an AU of 1. Informed consent was obtained from all individuals and their guardians, as described previously [26]. Ethical approval was obtained from the Ethical Review Committee at the Kenya Medical Research Institute and the Human Investigations Institutional Review Board at Case Western Reserve University and the University Hospital of Cleveland.

Samples of serum from eight volunteers experimentally immunized by the bites of irradiated, infected mosquitoes were obtained from W.O. Rogers at the US Naval Medical Research Center (Rockville, Maryland); and from U. Krzych at the Walter Reed Army Institute of Research (Washington DC). Upon challenge with bites of *P. falciparum *infected, non-irradiated mosquitoes out of the eight volunteers five were completely protected (W. Rogers and U. Krzych, personal communication).

### Immunoblot analyses

Purified GST-MB2 recombinant proteins (~50-100 ng) were resolved by SDS-PAGE in a 12% polyacrylamide gel and transferred onto a nitrocellulose membrane. The membrane was incubated with the human plasma or serum diluted 1:100 in Tris-buffered saline (TBS) + 0.05% Tween-20 (TBST) for two hours at room temperature. Following washing with TBST, the filters were incubated in HRP-conjugated anti-human IgG (CalBiochem, San Diego, CA) diluted 1:80,000 in TBST and the enhanced chemiluminescence (ECL) system (Amersham Biosciences Corp, Piscataway, NY) used for detection. Rabbit anti-GST antibodies were used as a positive control and normal human serum was used as a negative control.

### Amplification and sequence of MB2 genes from diverse parasite populations

*Plasmodium falciparum *genomic DNA extracted from laboratory-maintained strains and field isolates from infected red blood cells was kindly provided by Dr. A. Lal, (Centers for Disease Control and Prevention, Atlanta). The nucleotides encoding the antigenic region of the B domain of *MB2 *(aa 1-317 amino acids) were amplified from genomic DNA by the polymerase chain reaction (PCR) using the following primers: 5'-ATGTTTCTAATATGGCGTTTG-3' and 5'-TCATTTTTATTTGAAGAATT-3'. Conditions for amplification included an initial DNA denaturation of 94°C for 2 min, followed by 30 cycles at 94°C for 20 s, 55°C for 20 s, and 60°C for 1 min. Genomic DNA (200 ng) of each lab strain was used as template in the reaction. The concentration of genomic DNA of field isolates used as template in the amplification reaction is not known due to the limited quantity of the sample. The amplification products were cloned into a TA cloning vector using the TOPO-PCR cloning kit (Invitrogen). Plasmid DNA was prepared from bacterial cultures and the inserts sequenced in both directions. Cloning and sequencing were repeated on amplification products obtained independently to ensure reproducibility of the sequence data. Alignment of the sequences was performed by the Clustal method using the Megalign program from the Lasergene computer software.

### Rabbit immunization and total IgG purification

Rabbit immunization procedures used to obtain polyclonal antibodies against MB2 recombinant proteins are described elsewhere [20]. For IgG purification, the ImmunoPure^® ^IgG Protein A purification kit was used following the protocol provided by the manufacturer (Pierce, Rockford, IL).

### In vitro inhibition of sporozoite invasion (ISI) assay

To determine whether antibody targeting a particular domains or sub domains of MB2 had an effect on the ability of sporozoites to enter hepatocytes, total purified IgG from sera obtained from rabbits immunized against three different regions of MB2 (MB2-B, -C, and -FA) and a positive control NFS1 monoclonal antibody [27] were used in ISI assays of cultured liver cells [28,29]. A human hepatoma cell line, HepG2-A16, [28] was used for the assay. Briefly 50,000 HepG2-A16 cells were seeded on eight-chamber plastic Lab-Tek slides (Miles Research) in supplemented minimal essential medium (MEM) as described [28]. The purified antibodies were added to a final concentration of 100 μg/ml, and HepG2-A16 cells were infected with 25,000 *P. falciparum *NF54 sporozoites. Slides were incubated at 37°C in 5% CO_2 _for three hours, washed twice with phosphate-buffered saline (PBS), fixed with cold methanol, and rinsed twice again with PBS. Sporozoites that had invaded hepatoma cells were visualized by phase-contrast light microscopy using immunohistochemical staining with a mouse monoclonal antibody (NFS1) [27] against *P. falciparum *CS protein. Slides were incubated with NFS1 (10 μg/ml) for 30 min at room temperature, followed by goat anti-mouse IgG peroxidase conjugate for 30 min at room temperature. The ISI assays were done in triplicate with pre-immune IgG used as the negative control and the anti-CS monoclonal antibody NFS1 used as the positive control.

## Results

### Recombinant protein expression and purification

**Figure 1:**
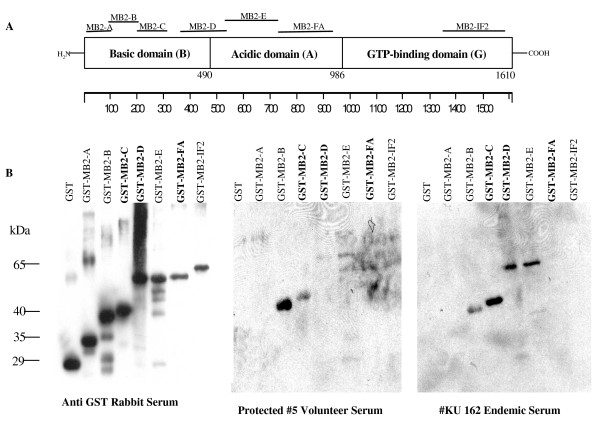
**Immunoblot analyses to assess the presence of antibody against MB2 recombinant peptides**. **A**, is a schematic representation of the *MB2 *protein sequence. The three domains, **Basic **(**B**), **Acidic **(**A**), and **GTP-binding **(**G**), are indicated as blocks with the amino acid junctions numbered below. The seven short horizontal lines represent the approximate extents of the polypeptides that were expressed as GST-fusion recombinant proteins. **B**, immunoblots of GST-MB2 recombinant proteins reacted with anti-GST rabbit serum (**Anti-GST**); or serum of a protected volunteer (**#5 volunteer**); or serum of a person living in a malaria-endemic area (**Endemic serum #KU 162**). Immunoblots were prepared in triplicate, and each lane contains 50-100 ng of purified GST-MB2 recombinant proteins. Recombinant proteins, MB2-C, MB2-D, and MB2-FA, listed in bold letters contain amino acid repeats. Approximate molecular weights of the fusion proteins are indicated in kilodaltons (**kDa**).

GST-MB2 recombinant proteins representing various regions of the coding sequence of MB2 were expressed in bacteria (Figure 1A). Bacteria transformed with plasmids carrying different regions of MB2 required different induction times and culture media for optimal yield (Table 2). The level of recovered protein varied greatly for each recombinant construct. The inclusion of 80-85 mM imidazole in the wash buffer and an overnight wash during isolation were essential in improving the purity of the recombinant proteins. All of the expressed proteins were soluble and thus purified readily from the cell-free bacterial lysates by one-step nickel column chromatography and were readily detected in an immunoblot using rabbit anti-GST antibodies (Figure 1B).

**Table 2:** Optimal conditions to express GST-MB2 recombinant polypeptides in *E. coli*.

**Name**	**Amino Acid Position**^**a **^**(Size)**	**Net Charge**^**b**^	**Media**^**c**^	**Induction Time**	**Expression Level**^**d**^
MB2-A	32-101, (70 aa)	+8.94	SB	1 hr 10 min	1-2 mg/L
MB2-B	95-206, (112 aa)	+10.18	LB	8 hrs	3 mg/L
MB2-C	200-316, (117 aa)	+2.58	LB	4 hrs	4 mg/L
MB2-F^e^(A-B-C)	32-316, (285 aa)	+21.91	SB	4 hrs	2 mg/L
MB2-D	355-546, (192 aa)	-0.72	SB	1-6 hrs	0.1-0.2 mg/L
MB2-E	538-773, (236 aa)	+8.23	SB	1-2 hrs	0.1-0.2 mg/L
MB2-FA	764-945, (182 aa)	-17.51	SB	3-6 hrs	2 mg/L
MB2-IF2	1337-1606, (270 aa)	+5.26	LB or SB	1 hr	0.5-1.0 mg/L

^a ^Position is based on the translation initiation methionine (1).

^b ^Calculated at neutral pH

^c ^LB, Luria Broth; SB, Super Broth. Media were made according to instructions in Sambrook *et al*.,[39].

^d ^Expressed as amount of purified protein per volume of IPTG-induced culture

^e ^Combined length of MB2-A+MB-2B+MB2-C

### Immunoblot plasma and serum analyses

All smear negative donors were exposed previously to *P. falciparum *as evidenced by the presence of anti-CS and anti-TRAP antibodies in their plasma (Table 3). The results of the immunoblot analyses obtained with the serum from a protected volunteer (#5, Table 3) and endemic plasma (KU162, Table 3), showed that both contain antibodies against the basic domain and some residual antibody reactivity was observed against regions of the acidic domain, whilst no antibody reactivity was detected against the GTP binding domain fragment as shown in Figure 1. Moreover, the regions of MB2 reacting with the immune sera are limited to the central region of the basic domain in particular MB2-B and MB2-C fragments. It would appear that serum from this protected (#5) individual reacts preferentially with MB2-B (non-repeat containing) and to a lesser degree with the MB2-C (repeat containing) region. In contrast the plasma from the endemic (KU162) region appears to have the inverse reactivity (i.e., MB2-C staining more strongly than MB2-B). Moreover, the endemic plasma also contained antibodies against the acidic domain fragments MB2-D and MB2-E. The protected sera (#5) had no detectable antibody against MB2-D, but some slight reactivity against MB2-E.

**Table 3:** Individual antibody responses to MB2 recombinant peptides of the B domain as determined by immunoblot analysis.

	**MB2-A**^**a**^	**MB2-B**^**a**^	**MB2-C**^**a**^			
**Volunteer serum**						
Volunteer #1 (protected)	- ^b^	++ ^b^	-/+ ^b^			
Volunteer #3 (protected)	-	++	-/+			
Volunteer #5 (protected)	-	++	-/+			
Volunteer #7 (protected)	-	++	-/+			
WRAIR #1 (protected)	-	++	-/+			
WRAIR #4 (not protected)	-	-	-			
WRAIR #5 (not protected)	-	-	-			
WRAIR #6 (not protected)	-	-	-			
						
**Kenyan serum** **Smear negative**	**MB2-A**^a^	**MB2-B**^a^	**MB2-C**^a^	**Age** **(yrs)**	**CSP** **AU**^d^	**TRAP** **AU**^d^
^c ^KU 036	-	++	+	29	1.77	2.35
KU 071	-	++	+++	18	1.24	2.08
KU 081	-	+	++	34	1.01	1.23
KU 069	-	+	++	18	0.55	1.31
KU 079	-	-/+	++	80	1.0	2.63
KU 083	-	-/+	+++	45	4.28	4.80
KU 163	-	++	-/+	18	1.75	1.46
KU 202	-	-	+	30	2.22	2.22
KU 205	-	-/+	-/+	44	1.81	1.17
						
**Smear positive, asymptomatic**						
KU 118	-	-	-	23	1.94	1.85
KU 044	-	+	-	6	0.75	0.79
KU 048	-	-	+	6	0.61	0.62
KU 076	-	-	-/+	6	1.29	0.72
KU 001	-	++	++	2	1.11	1.43
KU 049	-	-	-	6	1.64	0.66
KU 025	-	-	-	6	0.42	0.26
						
**Smear positive, symptomatic**						
KU 064	-	-	-	27	1.38	2.40
KU 070	-	+	-	18	1.41	1.59
KU 157	-	+	+++	20	1.64	1.37
KU 158	-	+	++	26	ND	ND
KU 165	-	-	-	26	0.34	0.10
KU 172	-	-/+	++	22	ND	ND
KU 199	-	+	-/+	19	ND	ND
KU 203	-	-	-/+	30	1.34	1.0
KU 207	-	-	+	19	ND	ND
KU 234	-	+	-/+	42	ND	ND
KU 162	-	+	+++	6	ND	ND
KU 062	-	-	-	6	0.87	1.85
KU 161	-	-	-/+	7	0.67	0.88
KU 041	-	-	-/+	6	1.62	0.71
KU 037	-	++	-/+	60	1.36	1.52
KU 072	-	-/+	-/+	24	0.59	1.0
KU 075	-	+	++	32	0.72	1.35
KU 080	-	-/+	+	34	0.87	1.50
KU 088	-	-	-	70	1.01	0.57
KU 145	-	+	+	40	3.01	2.56
KU 155	-	-	++	37	0.53	0.38
KU 174	-	+	+	22	2.27	1.90
KU 183	-	-/+	++	23	0.30	0.36
KU 067	-	-	+	6	1.90	2.95
KU 084	-	++	+	2	0.40	1.277

^a ^Peptides are described in Figure 1. ^b ^- no detectable band, -/+ weak band but visible, + weak band, ++ moderate to strong band and +++ intense band on western blot. ^c ^KU= serum obtained from individuals living in Kenya ^d ^AU = arbitrary units, see Methods for definition. CSP = circumsporozoite protein; TRAP = thrombospondin related adhesive protein.

ND = not determined

The initial immunoblot analysis showed that the protected volunteer (#5) serum recognized principally the two regions in the basic domain, and these regions were recognized differently by the endemic (KU 162) plasma. To determine if this differential antibody recognition correlated with the different immunity observed between protected volunteers and the status of intermittently-exposed individuals living in area of seasonal transmission, the immunoblot analysis was expanded to include plasma obtained from a total of forty one individuals living in an area of seasonal malaria transmission who were blood-smear negative for *P. falciparum *(n = 9), blood smear positive but asymptomatic (n = 7) or blood smear positive and symptomatic (n = 25). Serum samples from eight irradiated sporozoite exposed volunteers, of which five had acquired sterile immunity, were also included.

Results of the expanded immunoblot analyses of the basic domain fragments MB2-A, -B and -C are summarized in Table 3. A total of 83% (34/41) of naturally exposed individuals had antibodies against the basic domain of MB2 and prevalence against MB2-C and MB2-B was 78 and 61%, respectively. The prevalence of antibody against MB2-B and -C in plasma from smear negative donors relative to smear positive donors was determined (MB2-B 89%, MB2-C 100%) and (MB2-B 53%, MB2-C 72%) respectively. The overall prevalence of antibody response against the repeat MB2-C region was greater than against the non-repeat, MB2-B region. Comparison between smear positive (n = 32) (parasitaemic) and smear negative (n = 9) (non-parasitaemic) individuals for the presence of antibody against MB2-B approaches significance (p = 0.066, Fisher's exact test). Minimal or no antibody reaction was detected against the amino-terminal MB2-A region. In comparison, the immunoblot result obtained with sera of irradiated-sporozoite immunized volunteers showed an association between the anti-MB2 antibody response and the immune status of the volunteers (Table 3). The five protected volunteers produced anti-MB2 antibodies that were directed preferentially against the non-repeat MB2-B region. In contrast, all three unprotected volunteers did not produce a detectable antibody response against MB2.

All nine smear negative individuals had antibodies to CSP and/or TRAP, demonstrating evidence of prior exposure to *P. falciparum *(Table 3). As compared to smear negative individuals, smear positive individuals who had plasma available for testing for antibodies to CSP and TRAP by ELISA had lower levels of antibodies to TRAP (smear positive, median AU 1.13, range 0.1 - 2.95, as compared to smear negative, median AU 2.08, range, 1.17 - 4.8, P = 0.02) but not CSP (smear positive, median AU 1.06, range 0.3 - 3.01, as compared to smear negative, median AU1.75, range, 0.55 - 4.28, P = 0.11). Smear positive individuals were also less frequently positive for antibodies to CSP (14 of 26 individuals tested) or TRAP (15 of 26 individuals tested) (Table 3).

### Polymorphism assessment of the antigenic region of the MB2 basic domain

**Figure 2:**
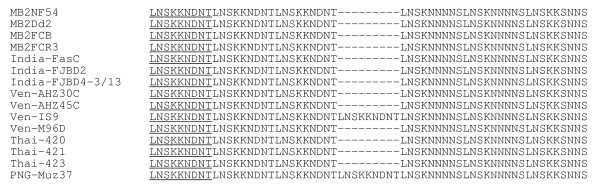
**Amino acid sequence alignment showing the size polymorphism in the repeat region of the MB2 gene from different laboratory strains and field isolates**. Amino acid positions 211 to 264 make up the repeat domain. Identical amino acids outside the repeat domain are not shown. Field isolates were surveyed from India, Venezuela (Ven), Thailand (Thai) and Papua New Guinea (PNG).

The nucleotide sequence encoding the first 317 amino acids of MB2 corresponding to the B-cell epitopes that were recognized differentially in the immunoblot analysis were determined. The nucleotide sequences have been deposited in GenBank with accession numbers AF454665, AF454666, AF454667, AF378132, AF378136 and XM_001351687. The translated amino acid sequence alignment of the antigenic region of MB2 from four laboratory strains and 11 field isolates collected from India (n = 3), Venezuela (n = 4), Thailand (n = 3) and Papua New Guinea (n = 1) show that the only variation observed is in the number of repeat units, two out of eleven isolates (Ven-IS9 and PNG Muz37) have 7 versus 6 repeats (Figure 2). No other polymorphisms were detected outside the repeat region

### In vitro inhibition of sporozoite invasion assay

The results showed that the IgG against the non-repeat-containing MB2-B peptide has the most potent inhibitory effect, 57% (Table 4). In contrast, the IgG against the repeat-containing peptide, MB2-C, exhibited only 18% inhibition. Moreover the IgG fraction against another repeat-containing but non-antigenic peptide, MB2-FA, conferred 33% inhibition.

**Table 4:** Evaluation of *in vitro *inhibition of sporozoite invasion (ISI) by rabbit antibodies against different regions of MB2

**IgG**	**SPOROZOITES/WELL**	**MEAN ± SD**^**a**^	**% INHIBITION**^**b**^
**MB2-B**			
PRE-IMMUNE	522, 514, 593	543 ± 35	0
MB2-B	205, 251, 238	231 ± 19	57%
**MB2-C^**c**^**			
PRE-IMMUNE	216, 312, 240	256 ± 40	0
MB2-C	252, 177, 198	209 ± 31	18%
**MB2-FA^**c**^**			
PRE-IMMUNE	472, 485, 438	465 ± 20	0
MB2-FA	263, 354, 306	307 ± 37	33%
			
**mAb NFS1**	11, 9, 6	8 ± 2	97%
**MEM^**d**^**Control	280, 350, 325	318 ± 29	0

^a ^SD, standard deviation

^b ^Inhibition expressed as % relative to pre-immune control culture (0%), and it was calculated as follows: 100 × [1 - (mean number of invaded sporozoites in test culture/mean number of invaded sporozoites in control culture)]. Pre-immune antibodies used as negative control.

^c ^These peptides contain amino acid repeat motifs.

^d^MEM Minimal essential medium.

## Discussion

MB2 is a novel sporozoite surface protein, that is present at several stages of the parasite lifecycle albeit in different sub-cellular locations as described earlier [20]. It is composed of three distinct domains, an amino terminal basic domain, a central acidic domain and a carboxyl terminal GTP binding domain as shown in the schematic Figure 1A. The surface localization of MB2 at the sporozoite stage provides a potential target for an antibody mediated response. Within the MB2 basic domain three overlapping polypeptides, and one non-overlapping polypeptide spanning between the basic and acidic domains, and three more polypeptides covering the remainder of the acidic domain and a C-terminal polypeptide were initially generated as GST-His-tagged sandwich fusions and used to immunize rabbits. The immune rabbit IgG was used in an inhibition of sporozoite invasion (ISI) assay. Antibodies against MB2 B inhibited sporozoite invasion (57%), whilst antibodies directed against MB-FA and MB2-C inhibited poorly (33% and 18% respectively) as shown in Table 4, suggesting that antibodies directed against this non-repeat region may be important in protection against infection. All five protected volunteers produced anti-MB2 antibodies preferentially against the non-repeat MB2-B region, and the ISI assay showed that rabbit anti-MB2-B antibodies are more effective than antibodies against other regions of MB2 at blocking sporozoite invasion of hepatocytes. It may be speculated that anti-MB2-B antibodies may contribute to the overall protective sterile immunity acquired by non-replicating metabolically-active sporozoite immunized volunteers. Immunoblot analyses of plasma from individuals in an area of highly seasonal transmission during a period of high transmission also demonstrated higher frequencies of anti-MB2-B antibodies among individuals who were not infected with *P. falciparum *as compared to those who were. However, these individuals also had higher levels of anti-MB2-C antibodies. In this preliminary limited study the sera were obtained at a single point in time and we cannot know whether either type of antibodies were associated with protection from infection in this population. It is very likely that the inoculum of infection in these individuals was much lower than that of the irradiated sporozoite-infected individuals. The data suggest that individuals with intermittent natural *P. falciparum *exposure have B cell responses that include regions of the MB-2 antigen recognized by those protected individuals exposed to irradiated sporozoites. The qualitative and quantitative antibody responses to the area most strongly associated with protection in immunized volunteers (MB-2B) may be impaired in individuals in areas of seasonal transmission. There was no clear relationship between malaria symptoms and presence of antibodies to a specific region (MB-2B or MB-2C), but since presence of antibodies was assessed at the time of disease, we can conclude only that symptomatic and asymptomatic individuals in this area are able to mount antibody responses to MB-2B and MB-2C. Further studies are required to characterize these responses in individuals who reside in areas of high-level, year-round *P. falciparum *transmission, and prospective studies required to assess the association of these antibodies with protection from infection and disease in endemic areas.

One of the striking observations is that protected volunteers were able to recognize preferentially the non-repeat MB2-B and to a lesser degree the repeat-including MB2-C and -D regions that most naturally exposed persons in malaria-endemic areas recognize. The significance of such a qualitative difference in the antibody response is difficult to assess due to the limited sample size. It is known that at the optimal radiation dosage required to induce sterile immunity, the weakened sporozoite is not able to develop completely in the hepatocytes [30,31]. Since the stage-dependent cellular localization of MB2 is accompanied by differential proteolytic processing [20], it may be that MB2 is aberrantly processed and is misdirected onto the surface of infected cells. Alternatively, B-cell response may be due to the exposure 'dose' of sporozoites. Protected volunteers received hundreds to more than a thousand bites of infected and irradiated mosquitoes in order to acquire sterile immunity [13]. In contrast, in malaria endemic countries, exposed individuals receive on average less than 200 infective bites per year [32], and in the highland area of Kenya where the endemic serum samples were collected, the number of infective bites is likely to be much lower than this [33]. It is possible that, the quantitative difference of inoculated sporozoites is responsible for the difference observed in antibody recognition against MB2 between protected volunteers and persons living in endemic countries. However antibodies against MB2-B were more frequent in those without parasitaemia than those with parasitaemia in the malaria endemic area, consistent with the findings in the protected versus unprotected volunteers.

The antigenic regions in the basic domain of MB2 were analyzed for potential amino acid polymorphisms. The nucleotide sequence of the *MB2 *gene was determined for field isolates obtained from different malaria-endemic regions of the world. It was expected that samples derived from non-overlapping locales would provide the greatest opportunity to detect sequence variation in *MB2*. The analysis of the primary structure of amplified MB2 DNA fragments from different isolates of *P. falciparum *showed that antigenic variation is unlikely to be a factor contributing to the different antibody response against MB2. Except for variation in the number of repeat units, the antigenic region in the B domain of MB2 is absolutely conserved among laboratory strains and field isolates collected from different parts of the world as shown in Figure 2. The amino acid sequence conservation may reflect a functional constraint of the B domain since it is not only exposed on the surface of the sporozoite but also translocated into the nucleus of blood-stage parasites[20]. In our *in vitro *ISI study, antibodies to this conserved, antigenic region of MB2 can inhibit sporozoite invasion of hepatocytes. If these antibodies play a role in protective immunity *in vivo*, the finding that the antigenic region of MB2 is highly conserved suggests that it might make a good target for immune attack by antibodies since most, if not all, *P. falciparum *sporozoites would be recognized.

The *Plasmodium *parasite is genetically complex, and based on the malaria genome sequencing projects [34] may have 5,000-6,000 genes. Its antigenic composition also is expected to be complex. Thus, the challenge in designing effective malaria vaccine(s) is to define the immunogenic molecules that are essential and the methods to present them properly to the immune system to induce the desired immune responses that protected the volunteers experimentally immunized with the non-replicating metabolically-active sporozoite vaccine. The MB2 protein possesses a number of molecular and immunogenic properties that indicate it is an intriguing candidate to complement current vaccine studies. Studies have shown that immunity to malaria also is mediated, at least partly, by cellular immune mechanisms [35]. In endemic areas, cytotoxic T lymphocytes (CTLs) from exposed individuals recognize epitopes in a number of pre-erythrocytic antigens of *P. falciparum*, and indirect evidence indicates that these CTLs may play a role in protective immunity [36-38]. Since MB2 was shown to be present in multiple developmental stages including the hepatic stage that can be recognized by CTLs, it will be important to obtain evidence that MB2 also is recognized by CTLs from *P. falciparum*-exposed individuals. The variation in the nature and strength of immune response observed in the endemic plasma samples may also be due to host factors such as MHC class I and II restriction. Further studies across multiple populations are needed to assess the type and strength of responses in individuals of differing genetic backgrounds. It is anticipated that as more novel parasite immunogens are characterized, the knowledge gained from studying them will help bridge the gap between recombinant and attenuated sporozoite vaccines.

The *P. falciparum *antigen MB2 is a multi-domain sporozoite surface protein [20]. In this study denatured recombinant peptide fragments MB2 were probed with immune plasma or sera resulting in the detection of antibodies directed against linear epitopes. Immunoblot analyses using serum of a volunteer protected by the exposure to non-replicating metabolically active sporozoites (#5) revealed that linear epitopes within the basic domain of MB2 are recognized strongly, whilst the acidic domain is recognized poorly. In comparison, plasma from a person living in an endemic region (KU 162) contained antibodies against linear epitopes within both basic and acidic domains. Antibodies against linear epitopes within the GTP binding domain were not detected in either set of samples. Moreover, sera from all the protected volunteers (non-replicating, metabolically-active sporozoite immunized) contain antibodies against MB2. In contrast, no anti-MB2 antibodies were detected in sera of non-protected (also non-replicating metabolically-active sporozoite immunized) volunteers. Furthermore, antibodies from the serum of protected volunteers recognized preferentially the non-repeat-containing MB2-B peptide, while the repeat-containing MB2-C peptide is recognized preferentially by the antibodies in the plasma of persons living in malaria-endemic areas. In addition, although the acidic domain also contains two amino acid repeat regions [20], serum from a protected volunteer showed minimal antibody reactivity against the acidic domain. In contrast, two regions of the acidic domain, one of which contains amino acid repeats, were recognized strongly by the antibodies in the endemic plasma (MB2-D & MB2-E). These qualitative results are interpreted to indicate that there may be one or more B-cell epitopes encoded in the non-repeat regions of MB2-B, that are more relevant to protective immunity than those encoded in the repeat regions. The results of the inhibition of sporozoite invasion assay suggest that antibodies directed against epitopes encoded in the non-repeat region (MB2-B) possess greater anti-parasitic activities than antibodies against epitopes encoded in the repeat-included region (MB2-C). The *in vitro *ISI assay showed that anti-MB2-B antibodies are more effective than anti-MB2-FA and anti-MB2-C antibodies at blocking sporozoites from invading the hepatocyte. Although ~40% of the parasites are still able to enter HepG2 cells, it is not known whether their intra-hepatic development is affected. The change of the cellular location of MB2 from the surface to the nucleus as the parasite lifecycle progresses from the sporozoite stage to the erythrocytic stage is consistent with the interpretation that it may have a function in the development of the parasite [20]. Thus, it is possible that anti-MB2 antibodies, although they partially block invasion of the sporozoites, they could also hinder the intra-hepatic development.

## Conclusion

The multi-domain MB2 protein is a sporozoite surface antigen identified on the human malaria parasite,*Plasmodium falciparum*. Analysis of serum from eight human volunteers that were immunized via the bites of *P. falciparum *infected irradiated mosquitoes, revealed five that developed immunity and were completely protected against subsequent challenge with non-irradiated parasite also had detectable levels of antibody against MB2. In contrast, in three volunteers not protected, anti-MB2 antibodies were below the level of detection. Moreover, anti-MB2 antibodies were detected in the plasma of 83% of the individuals living in a malaria endemic area of Kenya (n = 41). Antibodies from protected volunteers preferentially recognized a non-repeat region of the basic domain of MB2, whereas plasma from naturally-infected individuals had antibodies that recognize regions of MB2 that contain a repeat motif. Furthermore, rabbit polyclonal antibodies targeting the non-repeat region of the basic domain conferred greater inhibition of sporozoites entry into HepG2-A16 cells *in vitro *relative to antibodies directed against the repeat regions. Sequence analysis of eleven field isolates and four laboratory strains showed that these antigenic regions of the B domain of the *MB2 *gene are highly conserved in parasites obtained from different parts of the world. A preliminary analysis of the human humoral response against MB2 indicates that it may be an additional conserved target for immune intervention at the pre-erythrocytic stage of *P. falciparum *life cycle.

## Competing interests

The authors declare that they have no competing interests.

## Authors' contributions

TVN constructed the recombinant plasmids, expressed and purified the proteins and carried out the affinity purification of the rabbit antibodies, immunoblot analysis and DNA sequencing analysis. JBS and PdlV carried out the inhibition of sporozoite invasion assay. CCJ co-ordinated the collection of serum and plasma via KMRI.

AAJ and ASK initiated the study and supervised TVN. All authors contributed to data analysis and drafting of the manuscript.
